# AI-Enabled Microfluidics for Respiratory Pathogen Detection

**DOI:** 10.3390/s25185791

**Published:** 2025-09-17

**Authors:** Daoguangyao Zhang, Xuefei Lv, Hao Jiang, Yunlong Fan, Kexin Liu, Hao Wang, Yulin Deng

**Affiliations:** 1 School of Medical Technology, Beijing Institute of Technology, Beijing 100081, China; 3120215952@bit.edu.cn (D.Z.); xuefeilv@163.com (X.L.); 3120225713@bit.edu.cn (H.J.); xxl12621@126.com (K.L.); manghaobit@126.com (H.W.); 2 College of Pharmaceutical Sciences, Zhejiang University, Hangzhou 310058, China; fanyl@zju.edu.cn; 3 MicroTech Medical (Hangzhou) Co., Ltd., Hangzhou 311100, China

**Keywords:** respiratory pathogens, artificial intelligence, integrated microfluidics, POCT, intelligent diagnostics

## Abstract

Respiratory infectious diseases, such as COVID-19, influenza, and tuberculosis, continue to impose a significant global health burden, underscoring the urgent demand for rapid, sensitive, and cost-effective diagnostic technologies. Integrated microfluidic platforms offer compelling advantages through miniaturization, automation, and high-throughput processing, enabling “sample-in, answer-out” workflows suitable for point-of-care applications. However, their clinical deployment faces challenges, including the complexity of sample matrices, low-abundance target detection, and the need for reliable multiplexing. The convergence of artificial intelligence (AI) with microfluidic systems has emerged as a transformative paradigm, addressing these limitations by optimizing chip design, automating sample pre-processing, enhancing signal interpretation, and enabling real-time feedback control. This critical review surveys AI-enabled strategies across each functional layer of respiratory pathogen diagnostics: from chip architecture and fluidic control to amplification analysis, signal prediction, and smartphone/IoT-linked decision support. We highlight key areas where AI offers measurable benefits over conventional methods. To transition from research prototypes to clinical tools, future systems must become more adaptive, data-efficient, and clinically insightful. Advances such as sensor-integrated chips, privacy-preserving machine learning, and multimodal data fusion will be essential to ensure robust performance and meaningful outputs across diverse scenarios. This review outlines recent progress, current limitations, and future directions. The rapid development of AI and microfluidics presents exciting opportunities for next-generation pathogen diagnostics, and we hope this work contributes to the advancement of intelligent, point-of-care testing (POCT) solutions.

## 1. Introduction

Respiratory infectious diseases, including COVID-19, influenza, and tuberculosis, continue to pose a significant threat to global public health due to their high incidence, ability to spread rapidly, and potentially severe clinical consequences [1,2,3,4]. Timely and accurate pathogen diagnosis is essential for effectively interrupting the chain of transmission, optimizing patient management and improving prognosis [5,6,7]. However, current mainstream diagnostic techniques, such as polymerase chain reaction (PCR), mass spectrometry, and microbial culture methods, generally face limitations in addressing respiratory pathogen detection in terms of sensitivity, assay throughput, and operational costs. PCR is susceptible to primer design limitations (especially for highly mutated viruses), sample contamination, and low viral load, and the process is cumbersome and time-consuming [8,9,10,11]. Mass spectrometry techniques are inadequate for the detection of low-abundance proteins, complicated pre-treatment and limited throughput [12,13,14,15,16]. The culture method, which is the gold standard, is difficult to meet the demand for rapid diagnosis of acute respiratory infections due to low sensitivity (especially for caustic bacteria and antibiotic-exposed samples) and being time-consuming (days or even weeks) [10,17,18,19,20]. Therefore, there is an urgent need to develop next-generation diagnostic technologies to break through the existing bottlenecks and achieve rapid, sensitive, high-throughput and cost-effective detection of respiratory pathogens.

Microfluidics offers a promising solution for building sample-in answer-out fully integrated diagnostic platforms due to its miniaturization, integration, low sample/reagent consumption, and potential high-throughput processing capabilities [21,22,23,24,25,26,27,28,29,30]. This technology is expected to significantly simplify operational processes, shorten testing time, and reduce costs. However, it still faces multiple challenges in the clinical translation of respiratory pathogen detection: the high viscosity or heterogeneity of complex biological samples (e.g., sputum, saliva, aerosols) can easily lead to microchannel clogging or interfere with target capture [27,31,32]; the difficulty in efficient enrichment and detection of low-abundance targets (especially in large-volume primary samples) in a microscale environment [31,32]; the complexity of microfluidic chip design and optimization; limited automation and integration of critical steps such as sample lysis and nucleic acid extraction [29,32]; and the challenge of efficiently analyzing high-dimensional, multimodal data (e.g., fluorescence images, electrical signals) generated by microfluidic systems, which calls for more efficient and intelligent approaches [33].

**Figure 1:**
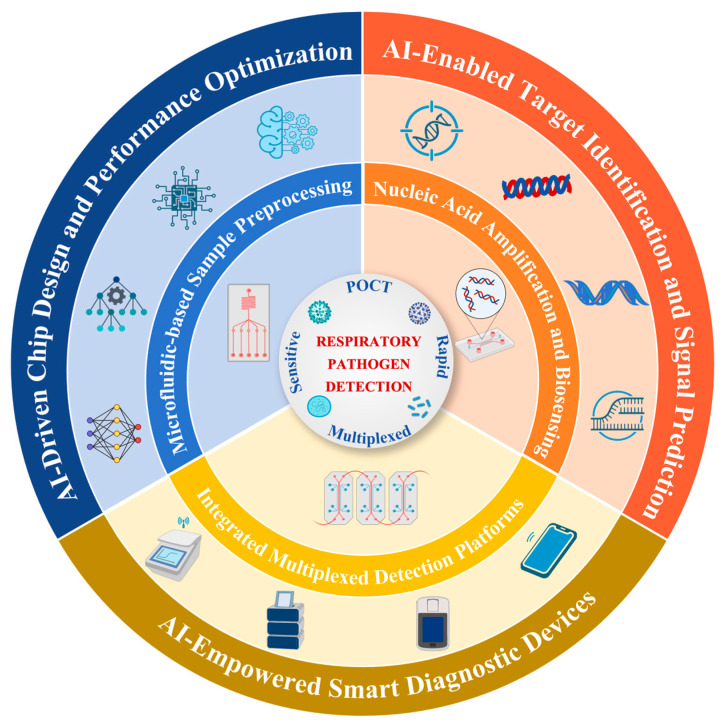
AI-enabled microfluidics for respiratory pathogen detection.

In recent years, the deep integration of Artificial Intelligence (AI), especially Machine Learning (ML) and Deep Learning (DL), with microfluidics has opened up new opportunities to address the above challenges and revolutionize the paradigm of respiratory pathogens detection (Figure 1). AI is able to optimize the hydrodynamic design and structure of microfluidic chips through data-driven modeling; empower intelligent pre-processing and target enrichment of complex samples; enhance the performance and reliability of platforms for highly sensitive detection based on nucleic acid amplification (e.g., digital PCR, LAMP, CRISPR) and biosensing (e.g., antigen–antibody, nucleic acid hybridization) high sensitivity assay platforms with high performance and reliability; enable intelligent analysis and interpretation of signals in high throughput multiplexed assays; and drive the development of portable, smartphone-integrated, POCT systems [33,34,35,36,37,38,39,40,41,42,43]. This synergistic effect is driving the birth of smarter and more powerful fully integrated microfluidic detection systems.

In contrast to previous reviews, which have either emphasized the application of AI in pathogen infection diagnosis and treatment from epidemiological and therapeutic perspectives [44], or focused on how AI enhances analytical and bioanalytical performance in general microfluidic systems [45,46], this review addresses a specific clinical application scenario. We present a comprehensive overview of the microfluidic technologies and AI for respiratory pathogen detection. Specifically, we highlight advances in microfluidic-based sample pre-processing, diagnostic approaches, and high-throughput multiplexed detection, which collectively enable automated, miniaturized, and sensitive POCT. In addition, we highlight AI-driven innovations—including chip design optimization, real-time signal interpretation, and intelligent control via smartphones or IoT frameworks—that significantly enhance system precision, throughput, and turnaround time. Future progress will depend on the development of adaptive computational frameworks that account for biological variability, the adoption of data-efficient and privacy-preserving learning models, and the integration of multimodal diagnostic data to support more robust and clinically actionable decisions.

This schematic illustrates an integrated framework of AI-enhanced microfluidic diagnostics for respiratory pathogen detection. The central goal is to achieve POCT with high sensitivity, rapid response, and multiplexing capability. Surrounding this core are three essential microfluidic modules: sample pre-processing, nucleic acid amplification and biosensing, and high-throughput detection units. Artificial intelligence is embedded in the outer layer, empowering three key aspects: chip design and performance optimization, target identification and signal prediction, and the development of IoT-based smart diagnostic devices, thereby enhancing system intelligence, throughput, and diagnostic accuracy.

## 2. Microfluidic Technologies in Respiratory Pathogen Detection

Microfluidics, with its unique miniaturization, integration, and precise fluidic manipulation capabilities, is becoming a central platform for the paradigm shift towards fully integrated, automated sample-in answer-out detection of respiratory pathogens. According to the main workflow stages of nucleic acid testing, this chapter focuses on two key domains—pre-processing and target enrichment of complex biological samples (Section 2.1) and microfluidic diagnostic approaches integrating nucleic acid amplification and biosensing (Section 2.2)—while Section 2.3 illustrates how integrated microfluidic platforms build upon these advances to achieve high-throughput and multiplexed detection. To address the complexity of respiratory samples such as viscous sputum or low-abundance aerosols, microfluidic platforms integrate chemical modification, magnetic bead manipulation, droplet microreactors, and physical separation to enable efficient lysis, purification, and target enrichment. In the core detection process, nucleic acid extraction is seamlessly combined with sensitive amplification techniques (e.g., digital PCR, LAMP, RPA, CRISPR-assisted assays) within miniaturized reaction units to achieve rapid and specific signal amplification, meeting POCT demands. Biosensing modules incorporating antigen–antibody or nucleic acid probes convert molecular recognition into real-time electrochemical, optical, or mechanical signals. Furthermore, high-throughput and multiplexed detection is achieved through parallelized designs such as centrifugal chips, droplet arrays, and spatially encoded microbeads, supported by modular architectures and advanced manufacturing. These integrated strategies collectively underpin the advancement and clinical translation of microfluidic-based respiratory pathogen detection.

### 2.1. Microfluidic Pretreatment for Complex Samples

Respiratory pathogen detection begins with processing complex biological samples (e.g., saliva, aerosols, sputum) that pose unique challenges: saliva contains inhibitors (e.g., mucins) that interfere with nucleic acid extraction, aerosols have ultra-low pathogen loads (<100 copies/mL), and sputum’s high viscosity risks microchannel clogging. Microfluidic pretreatment addresses these issues by integrating selective target capture, automated purification, and anti-interference design—with technologies tailored to the physical/chemical properties of each sample type. The following subsections detail sample-specific solutions, including magnetic bead-mediated nucleic acid extraction for saliva, inertial/electrostatic enrichment for aerosols, and inhibitor-removal strategies for sputum—all designed to ensure high-purity, high-yield target preparation for downstream detection.

#### 2.1.1. Saliva Sample Processing

**Figure 2:**
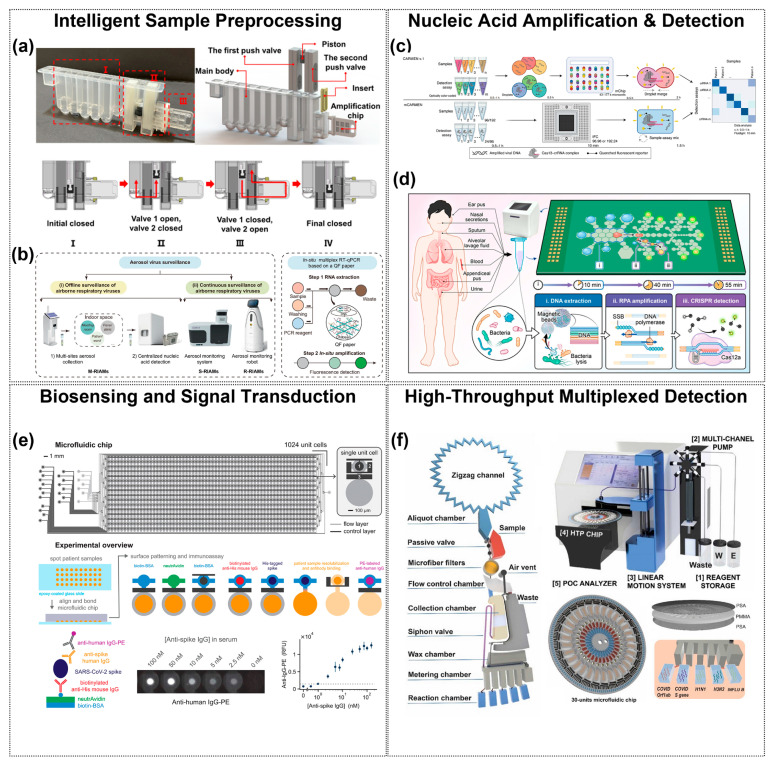
Microfluidic contributions at every stage of the respiratory-pathogen diagnostic. (**a**,**b**) Intelligent sample pre-processing cartridges integrate collection, lysis, purification, and metered transfer within a sealed chip, minimizing contamination and hands-on time. (**a**) shows the integrated cartridge structure with (I) magnetic-controlled nucleic acid extraction, (II) valve–piston fluidic control, and (III) a PCR chip; steps I–IV illustrate the valve–piston actuation enabling sealed and metered transfer. (**b**) shows three airborne-virus monitoring systems. Copyright 2023, Elsevier [48]. Copyright 2024, Nat. Commun. [54]. (**c**,**d**) On-chip nucleic-acid amplification and real-time detection architectures confine reactions to nanoliter volumes, accelerating kinetics and reducing reagent use. Copyright 2022, Nat. Med. [61]. Copyright 2025, American Chemical Society [62]. (**e**) Multiplexed biosensing and signal-transduction arrays route fluids through thousands of addressable microchambers, enabling parallel antigen/antibody or nucleic-acid assays with high analytical sensitivity. The two-layer chip contains 1024 unit cells, each with an immunoassay chamber (**top**) and a spotting chamber (**bottom**). Fluid control is achieved by button (1), sandwich (2), and neck (3) valves. Copyright 2021, PNAS [67]. (**f**) High-throughput read-out modules merge microfluidic partitioning with automated fluorescence acquisition, providing quantitative, multi-target results suitable for clinical decision-making. Copyright 2024, Elsevier [46].

Microfluidics has formed a multi-dimensional technology system for automated pre-processing and target enrichment of complex samples (e.g., saliva, aerosols) in respiratory pathogen detection. In saliva sample processing, integrated microfluidic systems use Virus Imprinted Polymer (VIP) technology to achieve target-specific enrichment. For example, Khan et al. [47] developed a six-channel microfluidic sensor using VIP(2-amino-1,3,4-thiadiazole) for selective H1N1 virus. The system integrates on-chip lysis and mixing for saliva detection with a low limit of 9 TCID50/mL. Fang et al. [48] designed a microfluidic cartridge system (Cartridge) to achieve efficient enrichment of salivary nucleic acids by magnetic nanoparticles (MNPs). After the sample and lysis buffer were injected into the microfluidic chip, the magnetic rod sleeve was vibrated and mixed at a frequency of 6 times/second to bind the MNPs to nucleic acids. The magnetic bar then transfers the MNPs to the wash buffer chamber, ultimately releasing the nucleic acids in an elution buffer at pH 10.4. This system can process 500 μL of saliva and complete nucleic acid extraction within 10 min with a sensitivity of 50 IU/mL (Figure 2a). It is worth noting that automated magnetic bead-based processing techniques are undergoing continuous innovation. The Integrated Microfluidic System with Electromagnetic Actuation (IMS) developed by Chiu et al. [49] represents a higher level of automation. The system utilizes aptamer-coated magnetic beads and electromagnetic actuation for virus capture, lysis, nucleic acid extraction, and RT-PCR. With only 50 μL of sample, SARS-CoV-2 and Influenza A/B viruses can be detected in 2 h with a detection limit as low as 200 copies/mL.

In addition, in saliva sample processing, the magnetic bead-mediated closed nucleic acid purification technique achieves fully closed operation through an oil phase environment to effectively avoid aerosol contamination. Specifically, the magnetic beads are driven by negative pressure to sequentially traverse the lysate, wash solution and eluent droplets to complete the capture and purification of nucleic acids [50]. In addition to magnetic beads, novel nanostructured materials have shown excellent enrichment potential. Jeon et al. [51] developed a microfluidic system with integrated biporous silica nanofilm that enhances nucleic acid enrichment via nano-vortices. The dual-pore structure boosts surface area and capture efficiency, enabling PCR-free detection with a 100-fold detection limit than conventional methods. For high viscosity samples such as mucin-containing sputum, the integrated Chelex-100 microfluidic thermal lysis method offers efficient nucleic acid extraction by chelating metal ions and inhibitors, followed by 95 °C lysis for 8 min. It significantly improves sample purity (OD260/280 from 1.18 to 1.79; OD260/230 from 0.77 to 2.17) and achieves high yields for both Gram-positive and Gram-negative bacteria (up to 196.96 ng/μL), with 98% concordance with the off-chip method [52].

Collectively, these studies demonstrate the rapid progress of saliva-based microfluidic pretreatment, spanning virus-imprinted polymers, magnetic bead actuation, nanostructured membranes, and chemical lysis methods. Their major advantages lie in reduced sample-to-extraction time, improved nucleic acid yield, and minimized operator intervention. However, the approaches remain challenged by variability in saliva viscosity and inhibitor content, as well as the difficulty of standardizing magnetic or nanomaterial performance across different clinical settings. Moreover, most systems have been validated only on limited sample volumes and selected viral targets, leaving questions about scalability and robustness for broader respiratory panels. To overcome these limitations, future strategies may integrate AI-driven real-time quality assessment to dynamically adjust microfluidic parameters (e.g., flow rate, channel geometry, mixing intensity) in response to saliva viscosity and inhibitor content. Hybrid enrichment schemes, such as combining magnetic nanoparticles with nanostructured membranes, could further enhance nucleic acid yield and purity. At the same time, machine learning models can be employed to compensate for batch-to-batch variability of magnetic or nanomaterials, enabling adaptive calibration across diverse clinical environments. Finally, modular chip designs that accommodate larger sample volumes and flexible respiratory pathogen panels will be essential for ensuring scalability and clinical robustness.

#### 2.1.2. Aerosol Sample Processing

Aerosol samples require a combination of efficient sampling and anti-interference techniques due to low viral loads and environmental interferences. For low concentration samples such as aerosols, Koo et al. [53] proposed a helical microfluidic preconcentration platform for respiratory pathogens (COVID-19, influenza A, RSV) in low-abundance aerosol samples. The APDMS-silanized channel enables ADH immobilization for efficient pathogen capture, processing up to 2.5 mL of aerosol samples with multi-step fluidic control to enhance enrichment. The RIAMs system developed by Liu et al. [54] utilizes a 400 L/min high-flow cyclone aerosol sampler to capture particles with a size > 0.8 μm by centrifugal force with a collection efficiency of nearly 100%. The sampler can be directly connected to the sample tube of the microfluidic chip to achieve “zero-cap” operation, avoiding cross-contamination, and the addition of filtering cotton significantly reduces the false-negative rate in response to environmental impurities (e.g., particulate matter in parking lot samples). Recent studies have further improved the sensitivity and applicability of aerosol sampling (Figure 2b). In addition, Jeon et al. [55] developed a microfluidic bioaerosol sampler with integrated inertial impact and electrostatic deposition, which was able to collect influenza A virus particles with an efficiency of up to 95%. Yang et al. [56] designed a Y-shaped sheath-flow microfluidic structure leveraging Dean vortex and inertial forces to efficiently separate 2 μm viral particles (95.99% efficiency) at 115 mL/min, avoiding protein oxidation inherent in electrostatic methods.

Microfluidic systems offer powerful capabilities for processing complex respiratory samples by integrating chemical modifications (e.g., VIP, ADH) for selective target capture, automated control via magnetic actuation and valve-piston mechanisms, and enhanced fluid handling through helical channels and vibratory mixing. The key innovation lies in their closed, contamination-free design with optimized inhibitor removal, enabling high-throughput, multiplexed analysis. However, variability in sample viscosity, inhibitor content, and pathogen abundance still pose significant challenges to pretreatment robustness and standardization. The application of AI, particularly in real-time quality assessment, adaptive parameter control, and automated workflow optimization, offers promising avenues to further improve the reliability and efficiency of this critical diagnostic step.

### 2.2. Microfluidic Diagnostic Approaches for Respiratory Pathogen Detection

After sample pretreatment, microfluidic diagnostic approaches convert pathogen-specific biomolecular interactions (nucleic acid, antigen–antibody) into measurable signals—with core goals of sensitivity, speed, and multiplexing. These approaches are categorized by recognition mechanism: nucleic acid amplification (the most sensitive, e.g., digital PCR, LAMP, CRISPR), molecular hybridization (enzyme-free, rapid), and antigen–antibody immunoassays (no amplification, POCT-friendly). Subsections below detail how each approach is optimized via microfluidic miniaturization, parallelization, and signal transduction—with emphasis on clinical applicability for respiratory pathogens (e.g., SARS-CoV-2, influenza A/B, Mycoplasma pneumoniae).

#### 2.2.1. Nucleic Acid Amplification-Based Detection

Microfluidic systems integrate biochemical reactions and signal detection into miniaturized, closed-form platforms, enabling rapid, sensitive, and multiplexed diagnostics for respiratory pathogens. According to their distinct biomolecular recognition mechanisms, these microfluidic diagnostic approaches can be classified into nucleic-acid amplification (e.g., digital PCR, loop-mediated isothermal amplification), molecular hybridization, and antigen–antibody immunoassay techniques.

Microfluidics provides an ideal platform for nucleic acid amplification by leveraging miniaturized reaction chambers, precise fluidic control, and efficient thermal management, enabling rapid, sensitive, and multiplexed detection of respiratory pathogens. Huang et al. [57] presents an integrated microfluidic PCR-array platform, which automates nucleic acid extraction and real-time PCR for multiplex detection of 21 respiratory pathogens. The system achieves high-throughput, contamination-free diagnostics with a detection limit of 10^3^ copies/mL, marking a key innovation in microfluidic–PCR integration for syndromic respiratory testing. Additionally, digital PCR combined with microfluidics further enhances detection sensitivity. Malic et al. [58] presents an innovative centrifugal microfluidic RT-ddPCR platform that enables fully automated, sample-to-answer detection of SARS-CoV-2 RNA. It achieves a detection limit of 0.1 copies/µL and 100% accuracy in clinical samples. Isothermal amplification technology (e.g., LAMP) shows significant advantages in paper-based microfluidic systems, which is especially suitable for multiple pathogen detection scenarios. The porous fiber structure of the paper-based material provides a specific surface area of up to 1.59 m^2^/g, which significantly enhances the nucleic acid adsorption efficiency [59]. Yin et al. [60] further utilized a 3D-printed microfluidic chip to integrate Reverse Transcription Loop-Mediated Isothermal Amplification (RT-LAMP) to detect SARS-CoV-2 in wastewater as low as 100 GE/mL within 60 min.

High-throughput nucleic-acid amplification combined with CRISPR technologies has been demonstrated by the mCARMEN platform, integrating Cas13 (RNA targets) and Cas12 (DNA targets) in spatially separated 192 × 24 reaction units, achieving 99.5% accuracy for SARS-CoV-2 variants in 2088 clinical samples, with a detection limit of 500 copies/μL (Figure 2c) [61]. In parallel, the MiND-DMF platform combined digital microfluidics and RPA-CRISPR-Cas12a to achieve automated, multiplexed detection of bacterial pathogens with a sensitivity of 100 CFU/mL within 60 min (Figure 2d) [62].

Centrifugal microfluidics provides a powerful platform for efficiently integrating and optimizing isothermal amplification of nucleic acids through its unique rotational dynamics and precise fluidic manipulation, significantly enhancing multiplexed detection capabilities. Suarez et al. [63] developed an injection-molded centrifugal chip integrating optical pH sensing for simultaneous RT-LAMP detection of SARS-CoV-2 and influenza A/B viruses, achieving detection limits down to 38 copies/reaction in under 48 min. The air-insulated microfluidic chip further takes the advantage of centrifugal control by accurately driving the sample distribution into 24 independent reaction chambers (volume of only 1.45 μL) by centrifugal force. It achieves rapid and sensitive detection (limit of 10 copies/reaction) for low-abundance pathogens, including Staphylococcus aureus, methicillin-resistant Staphylococcus aureus, and Mycoplasma pneumoniae, demonstrating 99.56% concordance with clinical qPCR results [64].

Nucleic acid amplification remains the backbone of microfluidic diagnostics, with PCR, digital PCR, isothermal methods, and CRISPR-based assays each contributing unique strengths. Integration with microfluidics has markedly improved throughput, automation, and sensitivity, with some platforms achieving near-single-copy detection and robust multiplexing. Nevertheless, these systems often face trade-offs between sensitivity and assay complexity, and are susceptible to inhibition from unprocessed clinical matrices. Additionally, the reliance on predefined primer sets or CRISPR guides raises concerns about adaptability to rapidly evolving viral genomes. While centrifugal and paper-based devices highlight the feasibility of low-cost and portable formats, their quantitative precision and robustness under real-world conditions require further validation. Incorporating AI-enabled primer/probe optimization and signal interpretation could help address these limitations, paving the way toward more resilient and adaptive amplification workflows.

#### 2.2.2. Biosensor-Based Microfluidic Diagnostics

Molecular hybridization diagnostics leverage sequence-specific recognition between nucleic acid probes and target genetic sequences, converting hybridization events into measurable electrochemical or optical signals, providing enzyme-free, rapid, and specific pathogen detection. Linear probes (LP), stem-loop probes (SLP), and stabilized analogs (locked nucleic acids, peptide nucleic acids) enhance specificity and stability. Hybridization signals are detected by electrochemical impedance spectroscopy (EIS), Förster resonance energy transfer (FRET), or voltammetry. Graphene oxide (GO)-mediated FRET sensing utilizes fluorescence quenching and recovery upon aptamer-target binding [65]. Integrated microfluidic systems achieve multi-channel and multiplexed detection through spatially separated hybridization sites, sample transport, temperature control, and on-chip detection electrodes. Liu et al. [66] demonstrated real-time fluorescent LAMP hybridization on a 10-channel microfluidic chip, detecting influenza A (H1N1), Mycoplasma pneumoniae, respiratory syncytial virus, and SARS-CoV-2 with sensitivity down to 10^3^–10^4^ copies/mL within 40 min.

Microfluidic antigen–antibody immunodiagnostics convert high-affinity immune binding events into measurable electrical, fluorescent, or colorimetric signals, providing rapid pathogen detection without nucleic acid amplification. Microfluidic nano-immunoassay (NIA) systems immobilize viral antigens (e.g., SARS-CoV-2 spike proteins) on electrodes, capturing specific antibodies in patient serum. Fluorescence amplification using secondary antibody conjugates (anti-human IgG-PE) achieves high sensitivity (98%) and specificity (100%) (Figure 2e) [67]. Molecularly imprinted polymer (MIP) sensors, exemplified by the H1N1 virus-imprinted polymer (VIP) sensor, generate shape-complementary binding cavities that alter electron-transfer resistance, detectable by electrochemical impedance spectroscopy [47]. Platinum nanoparticles (PtNP)-labeled immunocomplexes catalyze the generation of gas bubbles that form a visible light signal. Colorimetric assays achieve semi-quantitative detection by enzyme-catalyzed substrate chromatography (e.g., alkaline phosphatase-catalyzed BCIP/NBT) in combination with smartphone RGB analysis [65,67,68]. Emerging fabrication strategies also broaden the scope of microfluidic biosensors. Rolling microneedle templating [69] and photonic crystal–based flexible membranes [70] enable pump-free fluid transport, optical enhancement, and wearable integration. While originally applied in wound care or biochemical monitoring, these approaches highlight the potential of low-cost, customizable platforms for future POCT for respiratory pathogen detection.

In summary, microfluidic platforms have significantly advanced nucleic acid amplification and biosensing technologies through engineering innovations such as droplet encapsulation, spatial compartmentalization, and centrifugal fluid control. These strategies enable rapid, sensitive, and multiplexed detection of respiratory pathogens within fully integrated “sample-in–result-out” diagnostic systems. Nonetheless, several challenges remain, including primer interference in multiplex isothermal amplification (e.g., LAMP) and reaction inhibition caused by complex biological matrices such as saliva and sputum, which may compromise assay sensitivity and increase the risk of false-negative results [63]. Moreover, variability in pathogen characteristics necessitates precise adjustment of amplification parameters and biosensing thresholds. To address these limitations, algorithm-driven optimization—such as reaction curve prediction and kinetic modeling—holds promise for customizing amplification workflows to accommodate target-specific dynamics. Additionally, integrating multimodal signal outputs from electrochemical and optical sensors through computational analysis may improve result interpretation and enhance diagnostic accuracy, particularly in heterogeneous clinical samples.

### 2.3. Integrated Microfluidic Platforms for High-Throughput Pathogen Detection

To address the diagnostic challenges posed by pathogen diversity and mixed infections in respiratory diseases, integrated microfluidic systems have been developed to enable high-throughput, multiplexed detection. These platforms combine automated sample processing with parallel nucleic acid amplification and signal readout, offering rapid, sensitive, and contamination-free analysis. 96-channel microfluidic chip described by Zhang et al. [71] was fabricated through a molding process to achieve batch detection of pathogens using the magnetic bead method. The chip enables nucleic acid extraction within 10 min with tube-level efficiency, while its preloaded oil phase prevents aerosol contamination, supporting downstream high-throughput detection of 21 respiratory pathogens. A Centrifugal Microfluidic Disk (CMFD) is a typical example of a high-throughput assay that distributes samples to multiple independent reaction pools by centrifugal force-driven fluid flow in preset channels. Nguyen et al. [46] reported an advanced centrifugal microfluidic platform designed for high-throughput diagnosis of pandemic respiratory viruses, including influenza A H1N1, H3N2 subtypes, influenza B and SARS-CoV-2. Its core innovation is the zigzag aliquot structure on the chip, which realizes ultra-fast and equal distribution of reagents to 30 chambers in a single addition, accelerating high-throughput RNA extraction (Figure 2f). The system performs up to 150 RT-LAMP reactions in parallel using lyophilized reagents and primers, enabling multiplexed detection of 30 clinical samples within 1.5 h.

Microfluidic systems enable simultaneous typing detection of multiple respiratory pathogens by integrating the differences in physical properties of magnetic/non-magnetic microbeads. For example, Microbead-encoded chips introduced by Hong et al. [72] achieved multiplexed influenza subtype detection (H1N1, H3N2, H7N3) by encoding beads with magnetic and size differences and reading results via quantum dot fluorescence, with limits of detection as low as 2.2–3.4 ng/mL. This technique avoids the dependence of traditional multiplex PCR on expensive equipment and professional personnel, and increases the degree of automation by means of a miniaturized detection device with integrated computer control interface and bidirectional syringe pump [73]. The MONITOR system and the Onestart system [74] further demonstrate seamless integration of real-time PCR arrays within microfluidic chips, offering “sample-in–result-out” detection for 8–21 respiratory pathogens within 85–90 min, with high accuracy, sensitivity, and full process automation. Digital microfluidic (DMF) platforms have also emerged as powerful tools for multiplex testing. One system [75] enabled simultaneous detection of 11 respiratory pathogens (Mycoplasma pneumoniae, Chlamydia pneumoniae, respiratory syncytial virus A, adenovirus, coronavirus HKU1, coronavirus 229E, human metapneumovirus, SARS-CoV-2, influenza virus A/B, and others) with a detection limit of 12–150 copies/test and achieved 99.85% accuracy, 93.33% sensitivity, and 100% specificity in clinical validation. In addition, Bai et al. [76] developed a DMF-based POCT platform for 15-pathogen syndromic testing from untreated respiratory samples, achieving 200–628 copies/mL sensitivity across 32 replicates within 80 min.

Modular and interchangeable microfluidic architectures improve flexibility and scalability. The modular “Sticker Toolbox” microfluidics supports mass production while reducing costs through standardized template patch combinations and user-defined flow channel structures [77]. The NanoPEIA (Nano Plasma Enhanced Isothermal Amplification) technology combines a nano plasma sensor with isothermal amplification to complete the SARS-CoV-2 detection within 6 min, with sensitivity up to a Ct value of <25 for clinical samples [78]. In addition, Mesa Biotech’s Accula platform [79] utilizes an “instrument + disposable kit” model, which is pre-packed with lyophilized reagents and multiplexed primers, allowing users to automate nucleic acid extraction, amplification, and detection by simply adding samples. The platform can detect influenza virus, respiratory syncytial virus and SARS-CoV-2 by replacing different kits.

However, current chip-based high-throughput multiplexed detection systems still face several key challenges that constrain their clinical translation and performance limits. The first is the contradiction between detection throughput and flexibility. The physical isolation of reaction chambers (e.g., centrifugal disks) and fixed coding strategies reduce cross-talk but also limit the upper limit of the number of targets that can be detected at the same time, making it difficult to flexibly respond to the need for rapid adjustments of emerging/variant pathogens or personalized test combinations. Models relying on pre-positioned lyophilized kits also sacrifice some of this flexibility. The second challenge is the complexity of clinical samples. The non-homogeneity and matrix effect of real-world respiratory samples (e.g., high-viscosity sputum, inhibitor-containing aerosol-enriched fluids) can easily lead to reduced efficiency of the reaction within the microfluidic chip (e.g., enzyme inhibition, primer interference), which significantly increases the risk of false-negativity, especially for low-abundance targets. Existing systems lack the ability to sense and adapt to abnormalities in sample quality and reaction process in real time. The third is the bottleneck of multiple signal resolution. With the increase in the number of targets in the co-test, the kinetic differences between multiple amplification reactions, non-specific signal crossover, and complex background noise (e.g., bubble interference, autofluorescence) make the accuracy and specificity of the result interpretation face a severe test. Traditional thresholding methods or simple image processing are difficult to efficiently mine the effective information in high-dimensional data, which can easily lead to misjudgment or loss of sensitivity. These challenges essentially stem from the lack of intelligence in the “design-sample adaptation-signal analysis” process, which makes it difficult to realize dynamic optimization and closed-loop control. Artificial intelligence technologies, especially machine learning (ML) and deep learning (DL), provide revolutionary tools to overcome these bottlenecks through a data-driven approach, empowering microfluidic systems to realize intelligent reconfigurable design, real-time sensing and feedback control of sample processing, and high-precision parsing and early prediction of complex multimodal signals, thus breaking through the existing performance ceiling. This breakthrough will promote the existing performance ceiling and promote the establishment of a truly intelligent, robust and clinically practical high-throughput multiplexed assay platform.

The diverse microfluidic strategies presented in this chapter, ranging from automated sample pretreatment to nucleic acid amplification, biosensing, and high-throughput multiplexed detection, are systematically summarized in Table 1. This comparative overview highlights representative technologies and their performance indicators, providing a clear perspective on the current state of microfluidic approaches for respiratory pathogen detection.

**Table 1:** Performance indicators of representative microfluidic technologies for respiratory pathogen detection.

Technical Domain	Main Technology/System	Performance Indicators	Reference
Pretreatment—Saliva	Magnetic nanoparticle cartridge	500 μL sample; nucleic-acid extraction in 10 min; LOD 50 IU/mL	[48]
	Electromagnetically actuated IMS	50 μL sample; detection in 2 h; LOD 200 copies/mL	[49]
	Biporous silica nanofilm enrichment	100× enrichment over conventional methods; PCR-free detection enabled	[51]
	Chelex-100 thermal lysis for viscous sputum	Improved nucleic-acid purity (OD_260_/OD_280_: 1.18→1.79; OD_260_/OD_230_: 0.77→2.17); 98% concordance with off-chip	[52]
Pretreatment—Aerosols	Helical pre-concentration microfluidics	LOD 10× lower than conventional methods; 1–1.5 mL samples robustly processed;	[53]
	RIAMs high-flow cyclone sampler	LOD 10 copies/mL; integrated with 400 L/min aerosol sampler achieving 0.83 copies/m^3^ resolution	[54]
	Inertial–electrostatic bioaerosol sampler	Influenza A collection efficiency up to 95%	[55]
	Y-shaped sheath-flow inertial separator	95.99% separation for 2 μm particles at 115 mL/min	[56]
Nucleic Acid Amplification	Microfluidic PCR-array platform	LOD 1000 copies/mL; fully automated, contamination-free	[57]
	Centrifugal RT-ddPCR	LOD 0.1 copies/μL; 100% clinical accuracy	[58]
	3D-printed RT-LAMP	LOD 100 GE/mL; detection in 60 min	[60]
	mCARMEN (CRISPR-Cas12/13)	192 × 24 reactions; LOD 500 copies/μL; 99.5% accuracy	[61]
	MiND-DMF with RPA-CRISPR	Sensitivity 100 CFU/mL; 98–100% specificity	[62]
	Centrifugal RT-LAMP disk	LOD 38 copies/reaction; detection in 48 min	[63]
	Air-insulated centrifugal chip	LOD 10 copies/reaction; 99.56% concordance with clinical qPCR	[64]
Biosensing	10-channel LAMP-hybridization chip	LOD 10^3^–10^4^ copies/mL; detection in 40 min	[66]
	Nano-immunoassay (serology)	Sensitivity 98%; specificity 100%	[67]
	VIP-based six-channel sensor	LOD 9 TCID50/mL for H1N1; detection in 2 min	[47]
High-throughput Detection	96-channel magnetic-bead chip	Nucleic-acid extraction in 10 min; downstream 21-pathogen panel	[71]
	High-throughput centrifugal RT-LAMP	30 chambers; 150 parallel RT-LAMP; detection in 1.5 h;	[46]
	Microbead-encoded multiplexing	Influenza subtypes (H1N1/H3N2/H7N3); LOD 2.2–3.4 ng/mL	[72]
	MONITOR real-time PCR array	LOD 0.78–6.25 copies/µL for eight pathogens	[74]
	DMF multiplexed PCR	11-pathogen panel; LOD 200–628 copies/mL; accuracy 99.85%; specificity 100%	[76]
	Nanoplasmonic enhanced isothermal amplification (NanoPEIA)	96-sample throughput; LOD 23.3–28.3 copies/mL; sensitivity 100%; specificity 92%	[78]

## 3. AI-Enhanced Microfluidic Detection Workflow

AI emerged as a formal research field in the 1950s, with the term coined at the 1956 Dartmouth Workshop, where John McCarthy defined it as “the science and engineering of creating intelligent machines” [80,81]. Early research focused on rule-based systems and symbolic reasoning, yielding seminal works like the Logic Theorist and Samuel’s checkers-playing program—the first publicly recognized machine learning system [82]. Over subsequent decades, AI experienced cyclical phases of optimism and stagnation, marked by breakthroughs in expert systems and machine learning interspersed with two “winters” [83]. The past decade has witnessed exponential growth in deep learning, particularly in self-supervised algorithms, recurrent neural networks, reinforcement learning, and pre-trained models [84]. While recent advancements in natural language processing (e.g., GPT models) have demonstrated unprecedented capabilities, achieving strong AI remains elusive due to fundamental limitations [85]. Current research now focuses on developing advanced systems, including artificial consciousness and general intelligence.

Machine learning (ML) encompasses techniques that autonomously identify patterns in data to predict outcomes or inform decisions under uncertainty [86]. Its three primary paradigms are supervised learning, unsupervised learning, and reinforcement learning. Supervised learning algorithms train on labeled datasets to map inputs to outputs, with well-known examples including decision trees, random forests, and support vector machines [87]. Unsupervised learning, conversely, discovers hidden structures in unlabeled data through clustering, topic modeling, and anomaly detection, offering advantages in reduced reliance on labeled data and automated feature engineering [88,89,90]. Reinforcement learning (RL) distinguishes itself through an agent’s trial-and-error interaction with an environment to maximize cumulative rewards, making it ideal for developing self-improving intelligent systems with minimal human intervention.

Deep learning (DL), a subset of ML, employs artificial neural networks to model complex patterns. Architectures such as convolutional neural networks (CNNs), recurrent neural networks (RNNs), and generative adversarial networks (GANs) have been widely applied in bioinformatics, intelligent control systems, and other domains [91,92].

In healthcare, AI has revolutionized diagnostics, drug discovery, patient monitoring, and clinical decision-making [93,94]. Notably, in respiratory medicine, AI addresses the global burden of diseases like COPD and tuberculosis (TB) by enhancing early detection and management [95,96,97]. AI algorithms now analyze chest radiographs, CT scans, pulmonary function test data, and even respiratory audio signals to improve diagnostic accuracy [98,99,100]. Large language models (LLMs) have shown promise in medical documentation, record summarization, and educational tools, though concerns persist regarding accuracy, interpretability, and ethical implications [37].

The integration of AI with microfluidic technologies has revolutionized respiratory pathogen detection, enabling rapid, accurate, and automated workflows. This section categorizes advancements in AI-enhanced microfluidic systems into three critical domains.

### 3.1. AI-Driven Chip Design and Performance Optimization

As a miniaturized platform for manipulating fluids at microliter to milliliter scales, microfluidic chips, whose channel design directly affects the fluid flow pattern, mixing efficiency, and reaction kinetics, are the core foundation for determining the performance (e.g., sensitivity, throughput, and stability) of the detection system [101]. Traditional chip design is highly dependent on empirical knowledge and iterative prototyping, which is a time-consuming and labor-intensive process and difficult to globally optimize [35,102]. The introduction of AI, especially machine learning and deep learning, provides a powerful data-driven approach to chip design and realizes the paradigm shift from empirical to intelligence-driven. In terms of performance prediction and optimization, machine learning significantly improves design efficiency and accuracy.

#### 3.1.1. AI-Driven Sample Pretreatment Structure

Microfluidic chip technology has demonstrated significant advantages in miniaturization and integration in sample pretreatment, providing a new paradigm for rapid and accurate detection. The design of microfluidic chips aims to precisely control fluids at the microliter or even nanoliter level, such as controlling the flow rate, direction and volume of the fluid, while integrating multiple functions such as mixing, separation and reaction. A reasonable design of the channel can help shorten the sample processing time, reduce the sample usage, and enhance the detection signal, etc., thereby improving the overall efficiency and sensitivity of the analysis. However, the optimization of channel structure relies on trial-and-error experiments, which leads to the problem of low efficiency.

Zhang et al. [103] employed machine learning techniques and interpolation algorithms to design inlet structures capable of generating custom concentration gradients with arbitrary properties. These methods help achieve more precise control over concentration distribution and hold significant potential for reducing labor and experimental costs. Hong et al. [104] introduced an inverse design method based on deep neural networks (DNN). This method aims to establish a mapping relationship between channel geometries and concentration gradients, where simulated concentration gradient values are input, and inlet pressure and sample concentration serve as output variables. These works demonstrate the potential of intelligent data sampling to improve deep learning model performance and suggest that similar approaches could be valuable for addressing inverse problems in microfluidics. These works demonstrate the potential of intelligent data sampling to improve deep learning model performance and suggest that similar approaches could be valuable for addressing inverse problems in microfluidics. However, Zhang et al.’s approach [103] requires retraining when the geometry is modified and becomes unreliable under high Reynolds number conditions, while Hong et al.’s method [104] is highly data-intensive and entails elevated experimental and computational costs. These limitations also reflect common challenges currently faced in applying AI-based inverse design to microfluidics.

#### 3.1.2. AI-Driven Micro-Droplet Generation

Microdroplet generation, as the core technology of microfluidic chips, provides an ideal platform for achieving ultra-high-throughput analysis (such as single-cell sequencing and drug screening), high-sensitivity detection with extremely low reagent consumption (such as digital PCR), and integrated control of multi-step complex reactions by dividing continuous liquid phases into discrete, picolitre-nanoscale microdroplet units. Garstecki et al. [105] conducted an in-depth discussion on the formation process of droplets and bubbles in microfluidic T-junctions, such as the rupture mechanisms of droplets and bubbles, the causes of pressure drop, and the prediction of the proportional relationship between droplet and bubble sizes. Menech et al. [106] describes the results of a numerical investigation on the dynamics of immiscible fluid stream breakup in a microfluidic T-junction’s confined geometry. It identifies three droplet formation regimes (squeezing, dripping, and jetting) for microfluidic emulsification processes. The squeezing mechanism is unique to microfluidic systems due to fluid confinement affecting interfacial dynamics, with the breakup process mainly driven by pressure build-up and weakly influenced by the capillary number. The dripping regime, though seemingly similar to the unbounded situation, is affected by the constrained geometry which modifies the droplet size scaling law. The jetting regime occurs at high flow rates or with low interfacial tension (high capillary number), similar to the unbounded case. However, the on-demand generation of high-quality (monodisperse, highly stable, and specific-sized) droplets highly relies on precise fluid control and an in-depth understanding of complex multiphase fluid dynamics. The main challenges currently faced include: the difficulty in real-time and precise control of droplet generation (extremely sensitive to changes in flow rate, viscosity, and interfacial tension); During the generation process, heterogeneous droplets or agglomeration phenomena are prone to occur. Customized droplet parameter design for specific applications (such as encapsulating single cells or specific biochemical reactions) is inefficient and costly to trial and error. In the process of cell encapsulation, the initial cell concentration plays a crucial role. Since cells are randomly distributed within the encapsulation system to a certain extent, Poisson distribution can well describe such a random distribution pattern. For example, when we are encapsulating cells in microgels or other carriers, knowing the initial cell concentration allows us to apply the Poisson distribution model to predict the probability of finding a specific number of cells in a given volume or area of the encapsulation matrix. This, in turn, helps us to optimize the encapsulation conditions to achieve a more uniform and desirable cell distribution, which is essential for subsequent applications such as cell-based therapies or tissue engineering studies. These challenges stem from the nonlinear and multi-parameter strong coupling characteristics of physical processes, which make traditional modeling and control methods inadequate and urgently require the introduction of AI technology. AI can predict and dynamically optimize control parameters in real time by learning from massive experimental or simulation data to stably generate target droplets. Intelligently identify and generate defects and automatically adjust them; Efficiently assist in exploring the optimal design solution, thereby pushing micro-droplet technology towards higher precision, greater intelligence and broader application space.

Agnihotri et al. [107] observed the droplet formation kinetics of T-junctions under different flow conditions under the extrusion mechanism (capillary number Cac < 0.015) by changing the agarose gel concentration, temperature (40, 50, 60 °C), and the flow rate ratio of the continuous phase to the dispersed phase (ϕ). Numerical simulation shows that the formation process of agarose droplets includes five stages: filling, necking, breaking, threading, and rupturing. Among them, the threading stage is an additional stage that occurs when there is a non-Newtonian dispersed phase. Furthermore, numerical simulation shows that the threading length is proportional to the flow velocity ratio ϕ, and has a complex relationship with agarose concentration and temperature [108].

**Figure 3:**
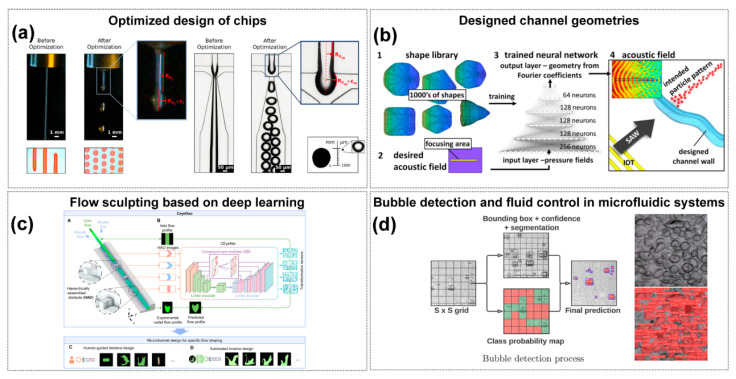
AI-Enabled Chip Design and Performance Optimization. (**a**) Fluid flow before and after control parameter optimization to form droplets. Copyright 2022, American Chemical Society [109]. (**b**) Deep learning-enabled acoustic field customization in microfluidic channels. (1) A shape library of thousands of acoustic field geometries is used to train a deep neural network, and (2) a target acoustic field is then defined as the input for design. Based on this, (3) the trained DNN outputs Fourier coefficients that determine the corresponding channel geometry, and (4) the modeled acoustic field in this geometry reproduces the desired focusing region and particle pattern. Copyright 2020, Sci. Rep. [110]. (**c**) AI-driven flow programming methodology developed to facilitate the design of microchannels that shape fluid materials into specific morphologies and combinations. (A) shows the microfluidic system, where core and sheath flows pass through sequential hierarchically assembled obstacles (HAOs) to achieve flow transformation. (B) shows the CEyeNet neural network with compound-eye modules for predicting outlet flow profiles in HAO-embedded channels. (C) shows human-guided iterative design, and (D) shows automated inverse design, together demonstrating the versatile flow programming capability of CeyeHao. Copyright 2025, the authors [114]. (**d**) Real-time computer vision feedback controls droplet/bubble dynamics and valve actuation, safeguarding fluidic reliability. The two images on the right illustrate the raw input (before processing) and the algorithm-processed output (after processing). Copyright 2024, *Mathematics* [123].

To achieve optimal droplet generation rates and sizes, Siemenn et al. [109] combined Bayesian optimization with computer vision to automatically identify stable droplet formation regions (Figure 3a). Deep learning iterated over 60 samples, converging on user-defined performance criteria, with the optimization process completed in just 2.3 h. This streamlined approach greatly enhanced the efficiency and accuracy of droplet behavior optimization. Raymond et al. [110] utilized deep neural networks (DNN) to design channel geometries capable of generating specific acoustic fields, enabling precise manipulation and arrangement of microparticles and cells, thus advancing research in the field (Figure 3b). Mahdi et al. [111] performed experimental studies and modeling on microdroplets generated in microfluidic systems using artificial neural networks to predict the dimensionless size of oil-in-water emulsion microdroplets. The “flow sculpting” technique involves shaping fluids into various geometries using columnar structures [112]. Different column arrangements alter fluid flow, creating a complex mapping relationship between fluid behavior and microfluidic design [113]. Yang et al. [114] developed an AI-enabled framework that integrates hierarchically assembled obstacles with the CEyeNet model to achieve programmable microchannel design. This approach broadened the range of attainable flow patterns, improved predictive accuracy, and significantly reduced computational cost, highlighting the role of AI in optimizing microfluidic chip architecture and performance. However, the method still relies on large-scale simulated training datasets, and its generalizability to more complex geometries and biological conditions requires further validation (Figure 3c). In other applications, Mekki-Berrada et al. [115] proposed a two-step framework for a machine learning-driven high-throughput microfluidic platform. This framework combines Gaussian process-based Bayesian optimization (BO) and deep neural networks (DNN), trained on 120 conditions of silver nanoparticle synthesis, to optimize synthesis performance. The approach remains constrained by the limited size and representativeness of the initial dataset, and its generalizability to other nanomaterial systems or more complex chemistries requires further validation.

Lashkaripour et al. [38] developed a tool named DAFD (Dynamic Design Automation of Fluids) to automate the design of flow-focusing droplet generators using machine learning. By analyzing 43 droplet generators, they studied the effects of different orthogonal dimensions and flow rates on droplet size and generation frequency. A neural network model, trained on a dataset of 998 data points, accurately predicted channel designs based on user-defined performance criteria. This method allowed for the estimation of droplet diameter and production rate errors to be within 10 μm and 20 Hz, respectively.

#### 3.1.3. AI-Driven Bubble Elimination

The ultimate goal of chip design is to realize high-performance and stable detection functions, and the stability of fluidic control (especially the bubble problem) is a key bottleneck to ensure that the design performance can be realized in actual operation. In such systems, fluid stability (absence of bubble interference) directly determines the accuracy, reproducibility and reliability of the assay. Bubbles are almost inevitable during sample loading, line connection or continuous operation, and their formation stems from hydrodynamic effects (e.g., negative pressure from rapid sample injection, high shear stress in narrow/curved flow channels to reduce gas solubility), precipitation of dissolved gases due to temperature variations, and the gas permeability of commonly used materials (e.g., PDMS) [116,117,118]. Gas bubbles can trigger localized pressure fluctuations, incomplete response, signal drift, false negative/positive results, and even blockage of the flow channel leading to equipment failure [43,116]. Traditional passive strategies mitigate the bubble problem by optimizing the flow channel structure and material properties, such as: increasing the inlet pressure [119], adding bubble capture traps [120], and modulating the surface wettability [121].

Artificial intelligence methods—including computer vision, machine learning, and deep learning—have begun to fill this gap [122]. Doganay MT et al. built a single-channel microfluidic model using transparent 3D microspheres to simulate bubbles, then benchmarked six ML and nine DL algorithms under varied imaging conditions [43]. Among ML models, random forest achieved 95.52% sensitivity, 82.57% specificity, and 97% AUC, while among mobile-compatible DL models, DenseNet169 reached 92.63% sensitivity, 92.22% specificity, and 92% AUC—maintaining accuracy above 0.84 on a smartphone-based POCT system—demonstrating AI’s potential for highly accurate bubble detection. Nizovtseva I et al. implemented a YOLOv9-based deep learning pipeline with high-speed video capture for real-time bubble segmentation and trajectory tracking in multiphase flows [123], and constructed a non-invasive platform to characterize gas–liquid mass transfer coefficients (Figure 3d). Beyond bubble detection, Xiao et al. [124] combined fuzzy logic, support vector machines, and principal component analysis to classify four canonical two-phase flow regimes (bubbly, slug, churn, and annular) in vertical microchannels by extracting texture features from dynamic images, enabling rapid and stable flow-pattern recognition for microscale fluid monitoring and control. AI has also been applied to fully automate fluid handling and bubble removal in immunoassays. Bhuiyan NH et al. developed a smartphone-operated, AI-controlled microfluidic immunosensing platform that uses lightweight ROI-cascading and conditional-activation algorithms to classify fluid states (including bubble presence and filling defects) in real time, then actuates on-chip micropumps and valves for precise liquid management [125]. An embedded bubble trap, triggered by AI-detected bubble locations, increases antigen–antibody contact area by 30–40%, greatly reducing false signals.

In summary, AI is deeply reshaping the core aspects of microfluidic chips from design to operation. On the design side, AI significantly improves design efficiency, accuracy and reduces the professional threshold through data modeling, performance prediction and reverse automation, promoting chip design from experience to intelligence. On the operation side, AI-driven bubble detection and intelligent fluid control solve the key stability problems in practical applications, forming a closed loop through real-time sensing, decision-making and execution, ensuring that the chip design performance can be stably realized under complex operating conditions, and improving the automation level and reliability of the system. The close integration of the two—i.e., using AI to optimize the design to improve the basic performance, and using AI to ensure the stability of the operation to realize the potential of the design—forms a complete closed loop of “AI-enabled chip performance optimization”. Together, they form a complete closed loop of “AI-enabled chip performance optimization”, which lays a solid foundation for building a high-performance and highly robust microfluidic system for respiratory pathogen detection. However, AI-driven design approaches still face challenges, with their model generalization capabilities limited by significant component differences and manufacturing lot fluctuations between laboratories [126]. Both Dressler et al. [127] and Hadikhani et al. [128] reported degradation in the accuracy of their predictive models across devices, which stemmed from manufacturing tolerances, PDMS aging, surface finish variations, connectivity methods, and even environmental factors (e.g., changes in fluid properties) have a significant effect on complex hydrodynamic behavior [35]. Models trained using a single laboratory data are prone to overfitting and it is difficult to build high-quality cross-organizational datasets, which constrains the generalizability of the models for application.

### 3.2. AI-Enabled Microfluidic Detection Methods

The on-chip respiratory tract pathogen detection technology based on microfluidic chips mainly includes signal generation and signal recognition. Among them, the generation of signals mainly relies on colorimetric, fluorescence or electrochemical methods. For instance, when metal ions produced in a reaction react with reagents to form colored compounds, a visible color change will occur. The target object is labeled with markers (such as fluorescent dyes, fluorescent proteins, quantum dots, etc.). When the target object exists and is subjected to specific excitation conditions (such as laser irradiation of a specific wavelength), it will emit a fluorescence signal of a specific wavelength. Or based on electrochemical processes, the target substance participates in REDOX reactions on the electrode surface, causing changes in electrical parameters such as current and potential, thereby generating detectable electrical signals, etc. For the different types of signals generated, corresponding detection equipment and technologies are adopted for detection. For optical signals, commonly used devices include fluorescence microscopes, photomultiplier tubes, charge-coupled device (CCD) cameras, etc. These devices analyze the condition of the target object by detecting parameters such as fluorescence intensity, color depth, and the wavelength of light absorption or emission. When detecting electrical signals, instruments such as electrochemical workstations are used to precisely measure the changes in electrical indicators like current, potential, and conductance, thereby determining the content, properties, and other information of the target substance. As an efficient tool, AI can play a significant role in the generation and detection of signals.

#### 3.2.1. AI-Enabled Bioinformatics Database

In actual testing samples, such as respiratory samples collected through throat swabs, nasal swabs, or other samples like blood and feces, the nucleic acids of pathogens (such as viruses and bacteria) often exist in extremely low copy numbers. Take the novel coronavirus as an example. In the early stage of infection or in the bodies of asymptomatic carriers, the viral load may be relatively low. It is extremely difficult to directly test such a trace amount of nucleic acid, and it is very easy to fail to detect it, that is, false negative results. Therefore, amplifying nucleic acids during nucleic acid testing is an extremely crucial step, which can ensure the accuracy and sensitivity of the test results as well as the effective quantification of target nucleic acids. It plays an indispensable role in numerous application scenarios such as disease diagnosis and pathogen monitoring.

In nucleic acid amplification, efficient primer design plays a decisive role in the detection results. At present, a variety of primer design algorithms have been developed for different isothermal amplification techniques.

The one-stop EasyDesign network design platform developed by Huang et al. integrates the design of recombinant polymerase amplification (RPA) primers [129]. RPA technology mainly relies on three key enzymes, namely recombinase, single-stranded binding protein and strand displacement polymerase, as well as a pair of specific primers, to achieve nucleic acid amplification. Recombinase can recognize and bind primers, and then search for complementary sequences in double-stranded DNA, promoting strand displacement reactions in the homologous regions of primers and template DNA, and forming local hybrid double-stranded structures. The single-stranded binding protein immediately binds to the single-stranded DNA that has been displaced, keeping it in a stable single-stranded state and preventing it from re-annealing into a double-stranded state. Strand displacement polymerase takes primers as the starting point and, based on the sequence of the template strand, continuously synthesizes new DNA strands along the template strand at around room temperature (typically with the optimal reaction temperature ranging from 37 °C to 42 °C) to achieve the amplification of the target nucleic acid. The team prepared a dataset containing 11,496 Cas12a detection data [130,131,132,133]. This dataset includes data from viral and bacterial sources with basically the same volume, involving zoonoses, human pathogens and animal pathogens. The data in the dataset can also be classified into mismatched, single-base mismatched and double-base mismatched data. Through the training and optimization of the CNN model, a high-performance CNN12ae model was obtained [133,134]. CNN12ae has demonstrated outstanding predictive performance in the design of crRNAs for various pathogens compared to traditional experimental methods, effectively reducing the workload of candidate crRNA screening.

In addition to the platforms mentioned above, there are also various software developed based on the principles of bioinformatics. PrimerExplorer V5 [135,136], developed by Eiken in Japan, is an online free loop-mediated isothermal amplification (LAMP) primer design software. Based on the LAMP reaction principle, it generates primer sets according to the input target sequence information [137]. NUPACK, developed by the Niles A. Pierce research group at the California Institute of Technology in the United States, is a software tool specifically designed for the design and analysis of nucleic acid structures, devices, and systems [138]. It can be used for structural analysis of nucleic acid complexes, nucleotide ensemble analysis, and nucleic acid structure design, etc. These tools facilitate primer design and nucleic acid research, and also lay the foundation for the development of AI algorithms and training data based on bioinformatics. By leveraging the vast amount of nucleic acid sequences and reaction data accumulated in bioinformatics, AI algorithms can be trained to enhance their performance in aspects such as the accuracy of primer design and the precision of nucleic acid structure prediction, thereby promoting the in-depth application of AI in microfluidic detection and broader biological detection fields.

#### 3.2.2. AI-Enabled Result Interpretation

In target identification and signal processing, AI algorithms extract pathogen-specific patterns from diverse microfluidic outputs (fluorescence, electrochemical, Raman). For instance, Khor et al. [39] employed a self-encoder to extract features of droplet deformations, combining it with a classifier to predict emulsion stability in confined channels with 91.7% accuracy. This capability is exemplified in flow-dynamics-based SARS-CoV-2 detection [139], where a Python algorithm (Python 3.7.4 and 3.8.2, Windows OS using Visual Studio) analyzes antibody-mediated immunoclustering effects on capillary flow kinetics. Real-time video processing via Otsu thresholding quantifies flow velocity changes, defining “time to constant flow rate” as a diagnostic parameter with 89% clinical accuracy [139].

Sun et al. [59] have addressed the application bottlenecks of POCT diagnosis in resource-limited regions, especially the time-consuming nature of traditional NAAT and its reliance on Cq value interpretation. The core of the research lies in the innovative combination of μPADs microfluidic chips and AI. The AI model section requires a key analysis: Transformer, as the core algorithm, takes in time-series fluorescence data collected by the chip (divided into raw gray values and G-channel values), and outputs the complete amplification curve of the prediction and the final value ratio. Here, it is necessary to emphasize the breakthrough of predicting 40 min results in 9 min and the high-precision indicators verified in clinical practice, slashing detection time by 78% while achieving 98.6% accuracy in clinical qPCR validation. However, the robustness of the model still depends on sufficient high-quality training data, may over-predict in low-copy or inhibited samples, and its generalizability across diverse pathogens and real-world clinical settings remains to be fully validated. Jiang et al. [40] leveraged optofluidic time-delay microscopy with CNN models to distinguish platelet aggregates from leukocytes at 10,000 cells/sec (96.6% specificity), while Muñoz et al. [41] applied random forests to identify fractal structures in LAMP reactions for label-free DNA quantification in sub-nanoliter droplets.

**Figure 4:**
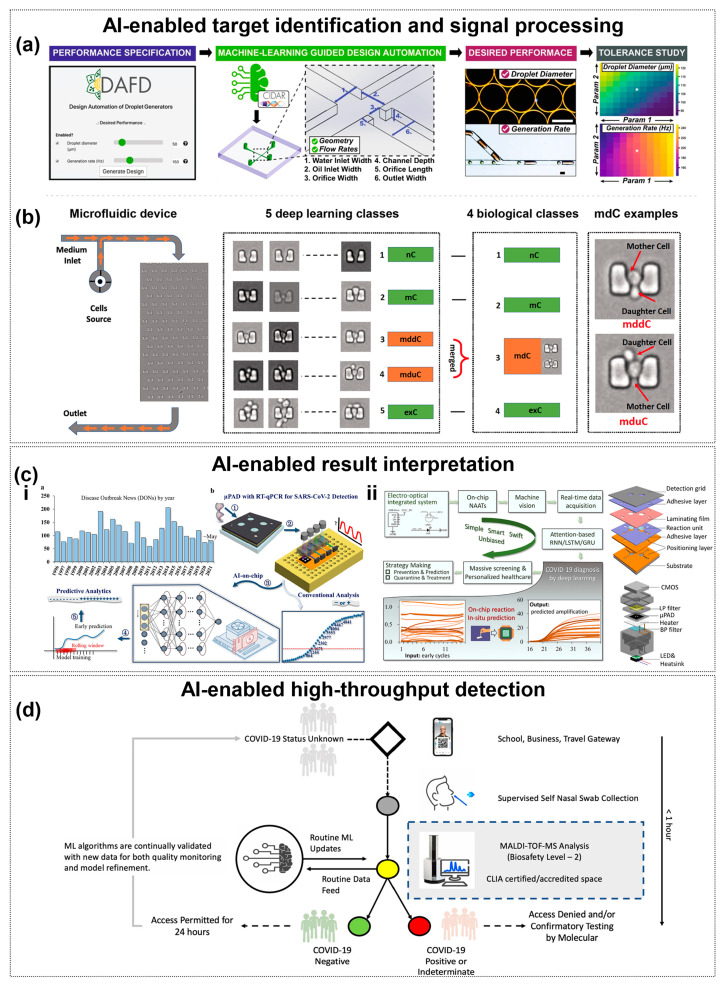
AI-Enhanced Pathogen Detection. (**a**) The machine learning enabled automated microfluidic design and control. User-defined performance targets are translated into channel geometry and flow rates, while the tool also predicts tolerance-induced deviations. The white pentagram indicates the nominal design condition (suggested design values) used as the reference point for tolerance analysis. Copyright 2021, Nat. Commun. [38]. (**b**) Image-based classification of cells captured in a microfluidic device with traps using deep learning. Copyright 2021, PLoS One [140]. (**c**) Neural Network-assisted target recognition and signal processing. (i) shows WHO-recorded disease outbreaks and the schematic of the proposed workflow. Step 1 to 5 describes the process: sample collection, RT-qPCR in µPADs, model training and validation, early time-series prediction, and final output. (ii) shows the attention-based GRU framework combined with µPAD and system integration, achieving higher accuracy, sensitivity, and specificity for real-time data interpretation. Copyright 2022, Fundamental Res. [143]. Copyright 2023, Elsevier [144]. (**d**) Conceptual model for near patient ML-enhanced MALDI-TOF-MS COVI D-19 testing. Copyright 2021, Sci. Rep. [152].

Artificial intelligence-driven design optimization addresses the detection challenges posed by viral mutations. Deep learning models, image-recognition techniques, and combinatorial optimization algorithms accelerate amplification prediction, simplify signal read-outs, and enhance target-sequence design precision: machine learning–based design automation tools like DAFD leverage neural networks trained on large-scale experimental data to predict droplet size and rate with high accuracy, enabling rapid, iteration-efficient stabilization of flow-focusing droplet generation (Figure 4a) [38], while CapsNet-augmented CNN models achieve robust classification of microfluidic cell division images by capturing both local features and spatial relationships. (Figure 4b) [140]. Gao et al. [141] presents an advanced machine-learning-assisted microfluidic nano plasmonic digital immunoassay designed for rapid, high-throughput cytokine profiling in COVID-19 patients. A key innovation is the CNN-based AI image processing, which enhances the speed and accuracy of nanoplasmonic signal detection by identifying individual silver nanocube (AgNC) scattering signals. Yang et al. [56] developed a rapid pathogen detection method by integrating microfluidic separation with spectroscopic analysis and machine learning (PCA and SVM). This AI-enhanced system processes high-dimensional spectral data to identify viral aerosols with 97.87% accuracy in under 30 min, comparable to PCR results.

Tran et al. [142] proposed a rapid phenotypic drug susceptibility detection (pDST) method that combines microfluidic chips, time-lapse phase contrast microscopy observation, and deep neural network (DNN) image analysis, targeting Mycobacterium tuberculosis bovine BCG (M. bovis BCG) and Mycobacterium smegmatis (M. smegmatis). Detection of anti-tuberculosis drugs such as rifampicin (RIF) and isoniazid (INH) can identify sensitive strains (slow-growing M. bovis—BCG) within 12 h, and for fast-growing M. smegmatis, growth rate differences can even be detected within 1 h. This method can also detect heterogeneous drug-resistant bacteria as low as 1%.

Sun et al. [143] combined a deep neural network (GRU model) with a paper chip to predict Ct values in only 20 PCR cycles (average error of 2.1%); Building on this concept, their subsequent study further advanced paper-based microfluidics by integrating an attention-based GRU network with real-time pixel-level fluorescence data. This approach exploited hidden reaction dynamics to predict amplification results after just 22 cycles, achieving over 97% accuracy, sensitivity, and specificity. The attention mechanism allowed the network to focus on relevant time-series features, outperforming conventional GRU and LSTM models and demonstrating robust cross-platform adaptability to clinical datasets. This highlights a powerful synergy of microfluidics and deep learning for portable, intelligent, and fast nucleic acid diagnostics [144] (Figure 4c). Wang et al. [145] developed the Fractal LAMP algorithm to achieve label-free detection by recognizing amplification by-products through bright-field microscopic images. The ADAPT (Activity-informed Design with All-inclusive Patrolling of Targets) system developed by Metsky et al. [146] utilizes a convolutional neural network (CNN) to predict CRISPR-Cas13a activity combined with a combinatorial optimization algorithm to design a guide RNA (gRNA) to maximize detection coverage of viral variants. Experimental validation shows that the gRNA designed by ADAPT improves the detection coverage of enterovirus type B (EVB) by >40% with zero cross-reactivity compared to the traditional conserved region design strategy. The system selects gRNA combinations through a submodular maximization algorithm, which ensures coverage of >95% of viral genome variants with 1–3 gRNA constraints.

Baltekina et al. [147] proposed a rapid antibiotic susceptibility detection (fASTest) method based on microfluidic chips, time-lapse phase contrast microscopy and single-cell imaging, which can complete the detection of common pathogenic bacteria causing urinary tract infections (UTI), such as Escherichia coli UPEC, within 30 min (including sample loading). This method monitors the growth rate of individual bacteria by customizing microfluidic chips (containing double-row cell traps that can capture bacteria as low as 10^3^ CFU/mL), and compares the differences between the drug treatment group and the control group. The response time to 9 anti-UTI antibiotics (such as ciprofloxacin CIP, ampicillin AMP) is the shortest, only 3 min. This method is also applied to detection of rare antibiotic susceptible bacteria in mixed sampled [148], blood infections [149] and clinical microbiology [150].

#### 3.2.3. AI-Enabled High-Throughput Detection

The breakthrough in the direction of high-throughput immunoassay is reflected in the synergistic innovation of microfluidic nano-chip and deep learning. The platform triggers fluorescence signals by antibody–antigen binding, and uses a custom Python algorithm to achieve 1024-cell parallel imaging analysis: firstly, the cell region is segmented to eliminate background noise, and the antibody titer (EC50) is calculated by fitting a saturation binding curve after intensity normalization [67]. An alternative technological path to feature extraction of sensing signals with a parallel strategy, Bae et al. [151] developed an integrated microfluidic system based on indium gallium zinc oxide (IGZO) field effect transistors (bio-FETs) for biosensors. The system utilizes Artificial Neural Networks (ANNs) to extract features directly from the raw output signals of the bio-FETs and perform accurate classification to achieve simultaneous discrimination of viral antigens (e.g., SARS-CoV-2 spike proteins) and antibodies in a single assay within 20 min. The method uses a pre-trained neural network and is fitness-trained with experimental data. The classification accuracies of antibody and spiny protein detection reached 98.85% and 93.22%, respectively, and the system detection limits were 1 pg/mL for antibody and 200 ng/mL for spiny protein. The technology significantly simplifies the process and reduces time and energy requirements compared to traditional complex methods such as PCR. In the COVID-19 assay, mass spectral data generated by MALDI-TOF-MS (>600 peaks in the range of 2000–20,000 Da) were processed by an automated machine learning platform, MILO. The platform employs ANOVA F-selector with Random Forest feature importance analysis to screen key mass spectral peaks, and combines Deep Neural Network (DNN) and Gradient Boosting Machine (GBM) models to achieve accurate classification of viral features. The optimal DNN model utilizes 487 mass spectral peaks (75% of the total number of features) to achieve 98.3% accuracy, while the GBM model achieves 96.6% accuracy with only 166 features (25%), which validates AI’s ability to reduce the dimensionality of the data while maintaining a high sensitivity (Figure 4d) [152]. Nevertheless, the approach inherently requires access to MALDI-TOF-MS hardware and associated sample preparation infrastructure, and its robustness may be compromised by domain shifts across laboratories or instruments, underscoring the need for broader cross-center validation before clinical translation. To address the problem of background noise and signal drift in microfluidic detection, a convolutional neural network (CNN) is used for data repair. For example, numerical discretization of fluorescence curves by Total Variation Regularization (TVR) algorithm eliminates signal discontinuities due to bubble motion or equipment fluctuations [63].

The above technological evolution shows that AI-driven signal processing is transforming from traditional endpoint detection to dynamic process resolution. The real-time data generated by microfluidic systems are transformed into early diagnostic indicators through three major technological routes: computer vision (flow pattern analysis), temporal modeling (amplification prediction) and image algorithms (fluorescence resolution). This paradigm change significantly improves detection efficiency—e.g., nucleic acid detection time is reduced by 78%—but clinical translation still needs to break through bottlenecks such as sample matrix interference (e.g., turbidity affecting flow pattern) and model generalization capability (e.g., low Ct sample prediction bias). Notably, these technological advances provide a technological foundation for the development of a distributed diagnostic model of home-based self-sampling and central laboratory analysis, which is expected to reshape the future surveillance system for respiratory infectious diseases.

### 3.3. AI-Empowered Integrated Portable Detection Device for Diagnostic Applications

Portable detection devices (POCT) are critical for resource-limited settings (e.g., rural clinics, field surveillance), but face challenges: smartphone camera variability, ambient light interference, and remote data management. AI addresses these by enabling smartphone-compatible signal analysis, IoT-based real-time control, and cross-device consistency—with two core integration paths: smartphone-integrated systems (low-cost, user-friendly) and IoT-linked platforms (remote monitoring, epidemiological tracking). Subsections below detail document-cited innovations.

#### 3.3.1. AI-Empowered Image Analysis for Smartphone-Integrated Diagnostic

The ubiquity and portability of contemporary smartphones enable microfluidic-based diagnostic platforms to transcend traditional spatial constraints. Their high-resolution digital imagers function as sensitive optical detectors, directly capturing colorimetric, fluorescence, or luminescence signals generated on-chip. Leveraging powerful embedded processors, smartphones can execute bespoke algorithms for real-time image processing, feature extraction, and quantitative analysis without the need for external hardware. An intuitive touch-screen interface further reduces the operational learning curve, while native wireless connectivity supports seamless data upload to cloud repositories, remote consultation, and integration with electronic medical records. Collectively, these features transform smartphones into compact, cost-effective, and user-friendly analytical hubs, extending POCT capabilities to resource-limited and field settings.

Smartphones for microfluidic testing have challenges such as camera localization, model differences, image distortions, and illumination condition variability, which constrain their clinical applicability. Deep learning (DL) models can overcome these technical bottlenecks by processing complex image features such as bubble morphology, size and distribution. For example, Wang et al. [153] developed a microfluidic dPCR system combined with smartphone imaging to detect SARS-CoV-2 RNA down to 3.8 copies/μL in a 20 μL reaction system, and its ellipsoidal pipette design generates a single, dispersed droplet to avoid cross-contamination, and the system is compatible with conventional PCR instruments. The ellipsoidal pipette design generates a single dispersed droplet to avoid cross-contamination, and the detection limit is one order of magnitude lower than that of traditional qPCR. The centrifugal microfluidic platform further integrates nucleic acid extraction and dPCR, realizing the closed-loop detection of “sample in—result out”. The SPyDERMAN system utilizes adversarial neural networks to process microfluidic chip images captured by smartphones for high-precision pathogen detection. The solution translates non-enzymatic signals (bubbles) into quantitative diagnostic results through end-to-end image analysis [154]. Traditional Convolutional Neural Networks (CNNs) rely on a large amount of expert labeled data and the model is severely restricted to a specific detection domain (domain-dependent). SPyDERMAN introduces the Adversarial Domain Adaptation (DA) strategy, which jointly optimizes the feature space and the classifier prediction to achieve fast system reconstruction across pathogens. The core of SPyDERMAN is to combine the limited labeled data The core of the approach is to reduce the inter-domain distribution difference by fusing limited labeled data (Source domain, e.g., specific virus images) with large-scale unlabeled data (Target domain, including simulated samples and synthetic images) for training. The method can achieve 100% detection accuracy when only a small amount of target pathogen data (e.g., SARS-CoV-2) is required.

By integrating high-resolution cameras and ambient light adaptation algorithms, smartphones can directly capture the physical or optical signal changes in microfluidic chips and realize real-time analysis using embedded AI models. For example, the VISTA system developed by Hardie et al. [155] uses an anti-neural network (SPyDERMAN) to process the image of oxygen bubbles generated by platinum nanoparticles in the microfluidic chip to realize the simultaneous detection of SARS-CoV-2 and HCV, and the detection accuracy of clinical samples is 93.3–95.45%. The system eliminates ambient light interference by optimizing the built-in light source of the smartphone and correcting the white balance of the image to ensure that the bubble morphological characteristics can still be stably identified in the non-laboratory environment.

Kim et al. [156] proposed a strategy for capillary flow analysis of peptide-modified particle-bacteria interactions, using flow rate variations within paper-based microfluidic channels to characterize the specific binding of different bacterial species. The video of capillary front movement was recorded by smartphone, and the flow rate parameters were extracted by customized Python script, which was combined with a support vector machine (SVM) classification model to achieve the identification of six bacterial species. This technique breaks through the limitations of traditional optical detection and only requires 2–6 s of flow rate data to be analyzed for classification. Kim et al. [157] further simplified the reagent system by adopting a “mix-and-use” strategy, whereby capillary flow rate is altered by the competitive binding of bacteria to lipopolysaccharide (LPS)/peptidoglycan. The smartphone video stream was processed by SVM to generate a multidimensional feature spectrum, and the mixed samples containing Gram-negative/positive bacteria were successfully differentiated. However, its performance remains limited by moderate accuracy in mixed samples, potential cross-reactivity of reagents, and the lack of mechanistic insight into the classification process, which restricts its clinical interpretability and broader applicability. Lu’s team [158] designed a microsphere encoding-decoding system (MMIP), which used multi-sized polystyrene microspheres as the signal carrier, and the smartphone was equipped with a Mobile Multi-Sphere Net (MMSN) deep learning model to realize multi-target detection of respiratory and viral viruses. virus multi-target detection. The model was optimized for microsphere classification and counting based on the YOLOv5 architecture, and the simultaneous quantification of FLUA, FLUB, and HPIV was completed in 30 min, with a detection limit as low as 0.14 pg/mL. Validation showed that the imaging differences between different brands of smartphones (iPhone/Motorola/Samsung) had no significant effect on the consistency of the model identification.

#### 3.3.2. AI-Empowered Image Analysis for IoT-Based Diagnostic

The integration of Internet of Things (IoT) technology serves as a critical enabler for intelligent, real-time pathogen detection across the entire diagnostic workflow. By seamlessly incorporating sensor nodes, wireless communication, cloud computing, and microfluidic platforms, IoT facilitates real-time monitoring of assay parameters, precise control of automated processes, instant transmission of diagnostic data, and cloud-based intelligent analysis. Furthermore, remote triggering of alerts or feedback mechanisms becomes possible, supporting proactive decision-making. This comprehensive integration not only enhances detection efficiency and system automation, but also reduces operational complexity and human error. Importantly, it overcomes spatial and temporal constraints, enabling POCT, large-scale data acquisition, and real-time analysis for population-level screening. Such developments mark a significant step toward a new paradigm of networked, intelligent, and highly responsive “smart healthcare” in pathogen diagnostics.

The computing power of smartphones further automates the control of the detection process. Yin et al. [159] designed a centrifugal microfluidic platform, called Mobile Multi-Sphere Net (MMSN), which is used for the microfluidic immune detection platform (MMIP) assisted by smart phones. SEDphone, to coordinate multitasking via an Android application: a Bluetooth temperature control module maintains dual temperature zones in the amplification and CRISPR detection zones, and a built-in CMOS sensor collects fluorescence signals generated by Cas12a cleavage, which is used by a machine learning algorithm to Automatic interpretation of five influenza virus subtypes (sensitivity 10 copies/μL). Clinical sample validation shows 100% positive/negative predictive value. This integrated design significantly improves operational efficiency, reducing time by 50% compared to traditional step-by-step testing.

**Figure 5:**
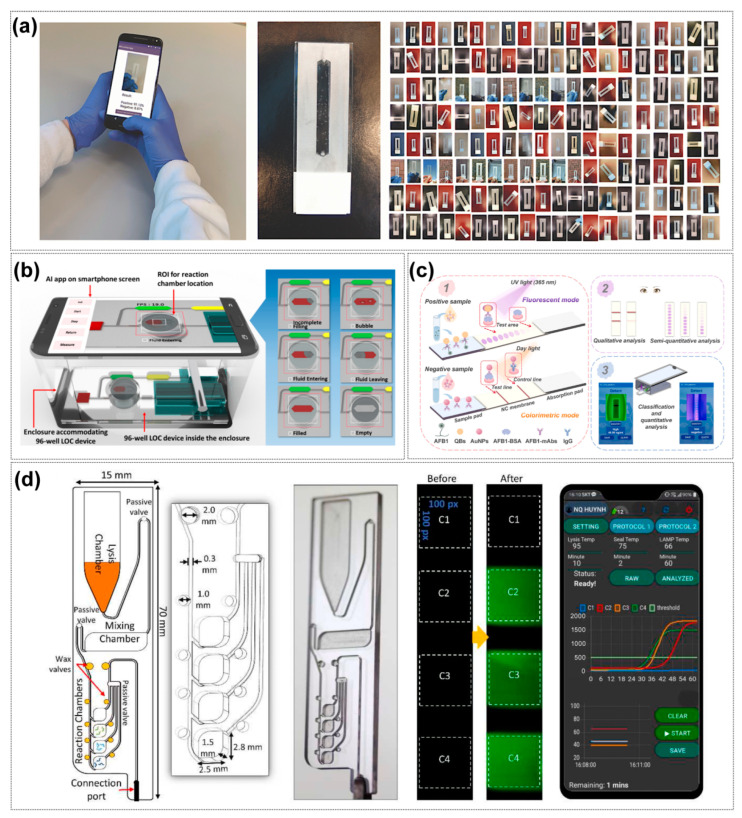
Smartphone and IoT-based applications. (**a**) AI algorithms integration and the tested microfluidic model system. Copyright 2024, Lab Chip. [43]. (**b**) an overview of the recognition patterns of liquids by the AI. Copyright 2022, American Chemical Society [125]. (**c**) A dual-modal immunochromatographic rapid detection system based on a deep learning strategy for smartphones. (1) illustrates fluorescent and colorimetric modes for preliminary sample assessment, (2) depicts qualitative and semi-quantitative visual analysis, and (3) shows smartphone-based quantitative detection with deep learning. Copyright 2025, Elsevier [161]. (**d**) Cloud-connected analytics fuse assay results with epidemiological engines, enabling POCT decision support and early outbreak forecasting. On the smartphone display, the four colored curves correspond to fluorescence signals from reaction chambers C1–C4, while the horizontal lines indicate the temperature profiles for lysis, wax sealing, and LAMP reaction. Copyright 2022, Elsevier [162].

Recently developed integrated platforms further combine artificial intelligence with Internet of Things (IoT) technology to automate and remotely monitor the entire process from sample to result. Nguyen et al. [160] developed a standalone microfluidic chip that integrates an Internet of Things (IoT)-based POCT device for efficient and cost-effective detection of respiratory viruses. The system automates the execution of RNA extraction, RT-LAMP amplification, and fluorescence detection, completing the sample-to-result diagnostic process in less than 70 min. The IoT-POCT module integrates a Raspberry Pi 4 computing unit for control and analysis, a CMOS sensor for fluorescence signal acquisition, an Arduino Nano microcontroller for temperature regulation mechanisms, and a solenoid valve for precise fluid control. Its core strength lies in its robust connectivity: wireless connectivity supports real-time data monitoring and remote results transmission, enhancing accessibility for telemedicine applications. A built-in touch screen allows users to view results instantly, and results can also be uploaded to a medical database for epidemiologic monitoring. Clinical validation showed that the platform has high sensitivity and specificity in detecting SARS-CoV-2, Influenza A and Influenza B without cross contamination. In summary, this IoT-POCT diagnostic platform provides a portable, smart and scalable solution for POCT and public health surveillance, especially in resource-limited settings. As illustrated in Figure 5, AI algorithms enable end-to-end microfluidic optimization through smartphone-integrated fluidic control, automated image-based diagnostics, and cloud-connected epidemiological forecasting, creating an intelligent workflow that bridges POCT with population-level pathogen surveillance.

The AI-driven innovations discussed throughout this chapter, spanning chip optimization, intelligent detection, and IoT integration, are systematically consolidated in Table 2 to provide a comparative overview of technical approaches and performance breakthroughs.

**Table 2:** Comparative Overview of AI-Enhanced Microfluidic Innovations in Respiratory Pathogen Detection.

Technical Domain	Core Innovation	AI Method	Performance	Advantages	Limitations	Reference
AI-Enabled Chip Design and Optimization						
Chip-design automation	DAFD workflow for flow-focusing droplet generators that predicts optimal channel geometry from user-defined specs	Feed-forward neural network trained on 998 data points	Diameter error ≤ 10 µm; frequency error ≤ 20 Hz	Rapid inverse design; reduces trial-and-error and prototyping cycles	Trained on limited datasets; transferability across materials/fabrication lots uncertain	[38]
Bubble detection & control	Real-time identification of bubbles in single-channel chip	Random Forest classifier on video frames	Sensitivity 95.5%, AUC 0.97; smartphone AUC > 0.84	Lightweight model deployable on phones; robust accuracy	Requires labeled videos; performance can degrade with lighting/optics changes	[43]
Gradient-generator design	Inverse mapping of channel layout to arbitrary concentration gradients	Machine-learning regression + interpolation	93.71% accuracy; 300× acceleration effect than conventional	Arbitrary gradient design; accelerates optimization of mixers/reactors	Needs retraining with geometry changes; interpolation fails at high Re	[103]
Multiscale droplet optimization	Automated search for stable droplet regimes	Bayesian optimization + computer vision feedback	Converged in 60 iterations; 8× faster than manual tuning	Sample-efficient tuning; minimal experiments	Setup-specific; may require retuning when fluids or geometry change	[109]
Acoustic field sculpting	Channel geometries that create user-specified standing-wave patterns	Deep neural network (DNN) inverse design	Programmable manipulation/assembly of particles & cells	Non-contact actuation; gentle handling of bio-samples	Sensitive to fabrication tolerances and acoustic hardware calibration; Requires specialized acousto-fluidics equipment	[110]
Flow design and inverse optimization	Programmable microchannel architectures using hierarchically assembled obstacles (HAO)	CEyeNet with receptive-field augmentation	Expanded diversity of flow patterns; high accuracy between simulation and experiment; significantly reduced computational cost vs. FEM	Coupling physical design rules with AI enables efficient, accurate, and scalable chip optimization	Requires extensive training datasets; generalization to unseen geometries and conditions remains limited	[114]
High-throughput synthesis	Two-step Gaussian-process BO + DNN for nano-particle microreactor	BO (global search) + DNN (local refinement)	Optimal silver-nanoparticle yield after 120 experiments	Efficient exploration of high-dimensional parameters; Demonstrates closed-loop optimization paradigm	Application-specific (nanoparticles)—indirect clinical relevance; Requires sufficient data/computation	[115]
Bubble segmentation & flow metrology	High-speed YOLOv9 pipeline for bubble tracking	YOLOv9 deep learning object detector	Non-invasive mass-transfer coefficient estimation	Real-time quantification of bubble dynamics; supports process modeling	Needs high-speed imaging + compute; heavier deployment footprint	[123]
Closed-loop fluid automation	Smartphone-operated immunoassay with on-chip pumps/valves	Lightweight CNN + fuzzy logic decision layer	cTnI LoD 0.98 pg/mL; 30–40% reduction in false signals	On-device QC and actuation reduce human error and artifacts; Demonstrates end-to-end sensing, decision, actuation	Adds actuator/firmware complexity; calibration required	[125]
AI-Enhanced Pathogen Detection						
Single-cell image analysis	On-chip CNN segments & classifies captured cells	Convolutional neural network	95% accuracy in cell-type identification	Label-free morphology-based analysis; integrates with microfluidics	Dataset/lab specific; generalization to new cell types limited	[33]
Real-time intelligent cell sorting	iIACS dual-membrane push-pull microfluidic sorter	CNN image classifier	2000 events s^−1^ with high purity	Real-time cytometry-like performance on-chip; Reduces manual gating	Specialized dual-membrane hardware; maintenance burden	[34]
Label-free droplet LAMP quantification	Detects fractal precipitate patterns in sub-nL droplets	Random Forest on bright-field images	Digital, dye-free DNA quantification at sub-nL scale	Eliminates fluorescent dyes/optics; reduces reagent cost	Pattern morphology sensitive to imaging/chemistry variations; Requires robust image standardization/pre-processing	[41]
Early amplification prediction	Forecasts PCR/LAMP endpoints from first 9 min of signal	Transformer with multi-attention	78% reduction in assay time; 98.6% clinical accuracy	Shortens turnaround time; earlier triage	May over-predict in low-copy or inhibited samples; Needs continuous, high-quality time-series fluorescence	[59]
Paper-based microfluidic nucleic acid testing	Early prediction of RT-LAMP results using real-time fluorescence on μPAD	Attention-based GRU network	Predicts 40-cycle results at 22 cycles with 98.1% accuracy	Low-cost paper platform with faster calls; Minimal hardware	Paper wicking variability can affect signals; Smartphone/ambient conditions add variance	[144]
Variant-resilient CRISPR design	gRNA sets that keep coverage as genomes mutate	CNN activity predictor + sub-modular optimiser	>95% variant coverage (≤3 gRNAs); >40% gain vs. baseline	Compact, mutation-tolerant assays for evolving viruses; Cuts wet-lab screening burden	Depends on up-to-date genomes; off-target risks persist	[146]
Simultaneous viral antigen/antibody assay	IGZO bio-FET array with on-chip microfluidics	Artificial neural network feature extractor	1 pg/mL Ab LoD; 200 ng/mL Ag; 98.9%/93.2% classification accuracy	Multi-modal serology on-chip; electronic readout	Microfabrication complexity; calibration drift	[151]
High-dimensional MALDI-TOF screening	AutoML selects key peaks for COVID-19 triage	DNN + Gradient-Boosting (MILO platform)	98.3% accuracy (487 peaks); 96.6% (166 peaks)	Leverages widely available MS platforms; Automated feature selection reduces manual curation	Requires MS hardware + sample prep infrastructure; Domain shift across labs/instruments can reduce accuracy	[152]
Smartphone-Integrated and IoT-Based Diagnostic Applications						
Smartphone imaging—cross-pathogen	SPyDERMAN adversarial DA converts bubble images to results	Domain-adaptation CNN	100% accuracy with few SARS-CoV-2 labels	Data-efficient; robust to cross-domain differences	Adversarial training can be unstable; requires careful tuning	[154]
Microfluidic Immunoassay	Platinum nanoparticle-catalyzed bubble signal readout + Ambient light adaptation algorithm	Adversarial Neural Network (SPyDERMAN)	Dual SARS-CoV-2/HCV detection: Clinical accuracy 93.3–95.45%; LOD:4000 copies/mL (SARS-CoV-2) LOD:2200 copies/mL (HCV)	Commodity cameras; resilient to ambient-light changes	LoD higher than PCR/CRISPR; relies on bubble kinetics	[155]
Paper-based Capillary Flow	Peptide–particle interaction-induced flow velocity changes	SVM classification	Six-bacteria identification; detection time 2–6 s	Ultrafast, equipment-light; no fluorescence/labels	Semi-quantitative; sensitive to viscosity/temperature	[156]
Competitive Binding Assay	LPS/peptidoglycan-bacteria competitive binding + “Mix-and-match” immobilization-free strategy	SVM multivariate analysis	Gram-negative/positive bacteria differentiation; mixed sample accuracy 75%	Reagent-sparse, rapid workflow; minimal immobilization	Moderate accuracy in mixtures; cross-reactivity possible	[157]
Microsphere Encoding-Decoding	Polystyrene microsphere signal carriers + Cross-platform smartphone compatibility design	Mobile Multi-Sphere Net (YOLOv5 architecture)	Triplex (FLUA/FLUB/HPIV) in 30 min; LoD 0.14 pg/mL; cross-smartphone consistency	High multiplex scalability via bead codes; very low LoD	Requires precise bead fabrication and optical setup; Potential code collisions/bleed-through in larger panels	[158]
Centrifugal Microfluidic-CRISPR	Bluetooth-controlled dual-temperature zones + Real-time CMOS fluorescence sensing	Machine learning classifier	Five influenza virus subtypes; sensitivity 10 copies/μL; 100%PPV/NPV; 50% time reduction	End-to-end task orchestration on phone; fewer steps	Requires custom electronics and power management; regulatory and cybersecurity considerations for connected devices	[159]
IoT-linked POCT platform	Edge-AI Raspberry-Pi device drives RT-LAMP cartridge	Embedded ML and cloud sync	3-virus panel in <70 min; >98% concordance	True sample-to-answer automation; remote QA and data aggregation	Depends on connectivity; privacy/security and device upkeep	[160]

## 4. Conclusions and Future Perspectives

In this review, we have systematically examined the synergistic integration of artificial intelligence and microfluidics, which is catalyzing a paradigm shift in respiratory pathogen detection. Conventional diagnostic methods are frequently hampered by limitations in speed, sensitivity, cost, and throughput. Microfluidic platforms offer a compelling solution by miniaturizing and automating complex laboratory procedures, enabling sample-to-answer systems with reduced reagent consumption and faster turnaround times. However, the full potential of microfluidics has been constrained by persistent challenges, including the processing of complex biological samples, the detection of low-abundance targets, the intricate process of chip design, and the analysis of high-dimensional data. Our analysis demonstrates that AI, particularly machine and deep learning, provides the critical tools to overcome these bottlenecks. AI-driven approaches are revolutionizing the diagnostic workflow by enabling the inverse design and performance optimization of microfluidic chips, enhancing the accuracy and speed of pathogen detection through intelligent signal processing, and driving the development of smart, connected POCT systems via smartphone and IoT integration. This convergence of AI and microfluidics is not merely an incremental improvement but represents a foundational leap towards creating diagnostic systems that are not only rapid, sensitive, and high-throughput but also intelligent, adaptive, and robust, providing powerful technological support for future clinical diagnostics and public health surveillance.

The future of AI in microfluidics will depend on the development of advanced algorithms capable of managing the complexity of biological data and adapting to emerging pathogens. While current AI-driven design approaches excel at optimizing fixed geometries for predefined conditions, their performance often declines in the face of the inherent variability of clinical samples—such as fluctuations in sputum viscosity or inhibitor concentrations—as well as manufacturing tolerances across devices. Future progress lies in creating truly “sample-aware” systems that integrate on-chip sensors to deliver real-time feedback on fluidic dynamics and sample properties. This data can power reinforcement learning algorithms to dynamically adjust fluidic parameters during operation, enhancing robustness across diverse sample conditions. At the same time, design optimization engines will autonomously select primers, guide RNAs, and probe sets that preserve detection coverage as viral genomes mutate. Together, these innovations will advance sensitivity, specificity, and generalizability across pathogen variants, paving the way for resilient and adaptive diagnostic platforms.

Second, a critical bottleneck in deploying AI-powered diagnostics is their reliance on large, high-quality labeled datasets for model training. The next frontier will move beyond conventional supervised learning toward data-efficient and privacy-preserving AI frameworks. Future research should prioritize the development of few-shot and zero-shot learning models, enabling rapid classification of novel threats without the need for extensive retraining. In parallel, implementing federated learning will be essential: this approach allows AI models to be collaboratively trained across multiple institutions on decentralized data, addressing the challenge of building high-quality, cross-organizational datasets while preserving patient privacy and data security. (e.g., a network of rural clinics sharing anonymized respiratory sample data without centralizing patient records) Such advances will help foster a more responsive and resilient diagnostic infrastructure, capable of supporting timely outbreak detection and coordinated clinical decision-making across diverse healthcare settings.

Third, the ultimate goal of diagnostic technology extends beyond simple pathogen identification toward delivering actionable clinical insights. The future lies in multimodal data fusion for predictive diagnostics. Current microfluidic platforms typically depend on a single data modality, such as fluorescence amplification curves or electrochemical signals. A next-generation AI-enhanced microfluidic system could capture and integrate multiple streams of information simultaneously—for instance, nucleic acid amplification kinetics, host immune-response biomarkers from immunoassays, and cellular morphological changes via on-chip microscopy. Advanced fusion models, such as Transformer architectures or graph neural networks, could analyze this high-dimensional data to not only detect co-infections with higher certainty but also predict disease trajectory, assess severity, and recommend optimal therapeutic strategies. By linking these multimodal outputs to clinical data through secure IoT frameworks, POCT devices could be transformed from simple detection tools into powerful platforms that assist clinicians in triaging patients during outbreaks, tailoring antiviral or antibiotic therapies, and monitoring recovery trajectories. For example, a portable microfluidic-IoT system deployed in a community clinic could automatically flag high-risk patients for hospital referral and simultaneously share diagnostic data with specialists for remote treatment planning.

In summary, the integration of AI and microfluidics is not merely reshaping respiratory pathogen detection—it is redefining the role of diagnostics in healthcare, shifting from reactive, single-target testing toward proactive, data-driven strategies that encompass both individualized patient care and population-level surveillance. By advancing adaptive algorithms, privacy-preserving data strategies, and multimodal fusion, this technology will enable faster responses to outbreaks, more precise treatment decisions, and greater access to high-quality diagnostics in resource-limited settings—ultimately reducing the global burden of respiratory infectious diseases.
